# Current aspects of targeting cellular senescence for the therapy of neurodegenerative diseases

**DOI:** 10.3389/fnagi.2025.1627921

**Published:** 2025-09-25

**Authors:** Ravindran Jaganathan, Ashok Iyaswamy, Senthilkumar Krishnamoorthi, Abhimanyu Thakur, Siva Sundara Kumar Durairajan, Chuanbin Yang, Dapkupar Wankhar

**Affiliations:** ^1^Preclinical Department, Faculty of Medicine, Royal College of Medicine Perak Universiti Kuala Lumpur, Ipoh, Malaysia; ^2^Mr. & Mrs. Ko Chi-Ming Centre for Parkinson’s Disease Research, School of Chinese Medicine Hong Kong Baptist University, Kowloon Tong, Hong Kong, Hong Kong SAR, China; ^3^Department of Biochemistry, Karpagam Academy of Higher Education, Coimbatore, India; ^4^Pritzker School of Molecular Engineering, Ben May Department for Cancer Research, The University of Chicago, Chicago, IL, United States; ^5^Molecular Mycology and Neurodegenerative Disease Research Laboratory, Department of Microbiology, Central University of Tamil Nadu, Thiruvarur, India; ^6^Department of Geriatrics and Shenzhen Clinical Research Centre for Geriatrics, Shenzhen People's Hospital, The First Affiliated Hospital, Southern University of Science and Technology, Shenzhen, China; ^7^Faculty of Paramedical Sciences, Assam down town University, Guwahati, India

**Keywords:** cellular senescence, senolytic drugs, neurodegenerative diseases, aging, DNA damage

## Abstract

**Introduction:**

Aging is a normal process causing deterioration in normal brain function and is inevitable. The aging process is described by the buildup of senescent cells and a decline in the ability to maintain essential homeostatic functions. Cellular aging represents a critical process where cells undergo cell cycle arrest in response to stress and neuronal damage. Many neurodegenerative disorders are closely linked to cellular senescence caused by oxidative stress, ROS generation, and DNA damage. Therefore, targeting cellular senescence is essential for the therapy of neurodegenerative disorders.

**Methods:**

This review outlines the understanding of cellular senescence, its role in the aging process, signaling pathways, autophagy, lysosomal biogenesis, and its contribution to various neurodegenerative disorders.

**Results:**

The findings highlight the relationship between cellular senescence and neurodegenerative disorders, emphasizing its pathological role. Current evidence indicates that senolytic drugs, notably phytochemicals such as dasatinib, quercetin, and fisetin, could serve as therapeutic approaches to target senescent cells and improve outcomes in neurodegenerative illnesses.

**Discussion:**

This review conclusively addresses the possibility of senolytic interventions for the treatment of neurodegenerative diseases. It will encourage researchers to identify novel compounds or phytochemicals that could be used as senolytic drugs for treating numerous neurodegenerative disorders.

## Introduction

The cellular phenomenon of aging is irreversible and is characterized by the arrest of cell division and induction of growth (Ogrodnik, 2021). Various exogenous and endogenous stressors, including telomere shortening, DNA damage, oxidative stress, and activation of oncogenes, can contribute to this phenomenon (Kowald et al., 2020). It is believed that this mechanism contributes to age-related illnesses, such as neurodegenerative diseases, as well as the ageing process itself (Kudlova et al., 2022; Hou et al., 2019). Accumulation of damaged proteins, inflammation, and cell death has been associated with cellular aging, which has been associated with the development of neurodegenerative disorders (Hou et al., 2019; Baker and Petersen, 2018; Hommen et al., 2022; Darios and Stevanin, 2020; Sarkar and Nazir, 2022).

The ability of aging cells in the brain to release pro-inflammatory chemicals like cytokines and chemokines is another factor that contributes to the deterioration of neurons and the advancement of neurodegenerative disorders (Baker et al., 2011; Gonzales et al., 2022). The accumulation of *β*-amyloid and tau proteins seen in Alzheimer’s disease (AD), coupled with the clustering of aging microglia and astrocytes in the brain, worsens neuroinflammation. The progression of the condition is predominantly influenced by the buildup of these *β*-amyloid and tau proteins (Stern, 2012; Liu, 2022). Similarly, the accumulation of senescent dopaminergic neurons and microglia has been accompanied by the pathogenesis of Lewy bodies and the neuroinflammatory response in individuals with Parkinson’s disease (PD) (Beraud et al., 2011; Han et al., 2020).

At present, exploring cellular senescence as a potential therapeutic target for addressing neurodegenerative diseases has increasingly received the attention of research focus (Guerrero et al., 2021). Current research has looked at the possibility of alleviating the symptoms of neurodegenerative diseases by using senolytics, which are pharmaceuticals or chemical compounds that selectively remove aged cells (Childs et al., 2015). In animal models of AD and PD, senolytic therapy has been demonstrated to enhance cognitive function and decrease neuroinflammation in both diseases (Zhang et al., 2019; Chinta et al., 2018).

In this review, we have investigated the impact of cellular senescence on the progression of neurodegenerative conditions such as AD, PD, amyotrophic lateral sclerosis (ALS), and Huntington’s disease (HD) (Lopez-Otin et al., 2013; McHugh and Gil, 2018). We have also discussed several markers, signaling pathways, and causes of cellular senescence, which include cyclin-dependent kinase (CDK) inhibitors p16, p21, retinoblastoma tumor suppressor protein (p-RB), high mobility group box 1 (HMGB1), lamin B1 (LMNB1), senescence associated *β*-galactosidase (SA-β-gal), senescence-associated secretory phenotype (SASP), mechanistic target of rapamycin (mTOR), autophagy, lysosomal biogenesis, ubiquitin proteasome system (UPS), mitophagy, DNA damage, telomere shortening, and oxidative stress (McHugh and Gil, 2018; Sahu et al., 2022; Lee et al., 2023). This review highlights the potential of senolytic phytochemicals, such as dasatinib, quercetin, and fisetin, as a therapeutic agents - targeting aging cells and reduce the symptoms of neurodegenerative illnesses (Bussian et al., 2018; Lagoumtzi and Chondrogianni, 2021). We aim to emphasize the necessity for conducting further research to identify innovative senolytic drugs and enhance the effectiveness of employing these agents in the treatment of neurodegenerative conditions.

## Cellular senescence

Cellular senescence is a permanent arrest of the cell cycle caused by factors like DNA damage, telomere shortening, activation of oncogenes, and oxidative stress (Baker and Petersen, 2018; Childs et al., 2015). Senescence is marked by the upregulation of several markers, including p16 and p21, retinoblastoma protein (p-RB), and SA—*β*-gal (Table 1) (Baker et al., 2011; Childs et al., 2015; Chinta et al., 2018). Cells undergo changes in many ways, including how genes are expressed, how chromatin is structured, and how they use energy during cellular senescence (Childs et al., 2015). Also, senescent cells often show a SASP, in which they release cytokines, chemokines, growth factors, and other molecules that can benefit or harm the cells and tissues around them (Guerrero et al., 2021; Childs et al., 2015). Senescence is a normal event that occurs in the human body. It helps with growth of tissues, healing wounds, and stopping tumours from growing (Guerrero et al., 2021). As individuals age, senescent cells can accumulate and perform a significant role in the onset of cancer, heart disease, and neurodegenerative disorders, all of which are associated with the aging process (Zhang et al., 2019).

**Table 1:** The key markers with high sensitivity and specificity involved in cellular senescence of the different organelles in *in vitro* and *in vivo* models.

Marker	Location	Role and other details
p16	Nucleus	Inhibitor of CDKs, upregulated in aging cells, associated with DNA damage and cell cycle arrest.
p21	Nucleus	Inhibitors of CDK, are upregulated in senescent cells, related to response to DNA damage and cell cycle arrest
p-RB	Nucleus	Retinoblastoma protein plays a crucial role in the regulation of the cell cycle by inhibiting the transition from G1 to the S phase, downregulated in senescent cells.
HMGB1	Nucleus and cytoplasm	HMGB1 released from the senescent cells triggers inflammation and immune response.
LMNB1	Nuclear lamina	The downregulation of LMNB1, a structural protein found in the nuclear lamina, in senescent cells results in changes to nuclear structure and function.
SA-β-gal	Lysosomes	SA-β-gal, an enzyme located in lysosomes, is a widely utilized marker for senescence.
SASP	Extracellular space	SASPs produced by aging cells influence inflammation and tissue remodeling.

### Markers

#### Signaling

The process of cellular aging is a complicated process that requires the participation of several signaling channels including the mTOR, autophagy, lysosomal biogenesis, UPS and mitophagy (Kudlova et al., 2022). The interplay between the mTOR signaling pathway and autophagy is critical in the context of neurodegenerative diseases such as AD and PD.

#### mTOR

**Figure 1:**
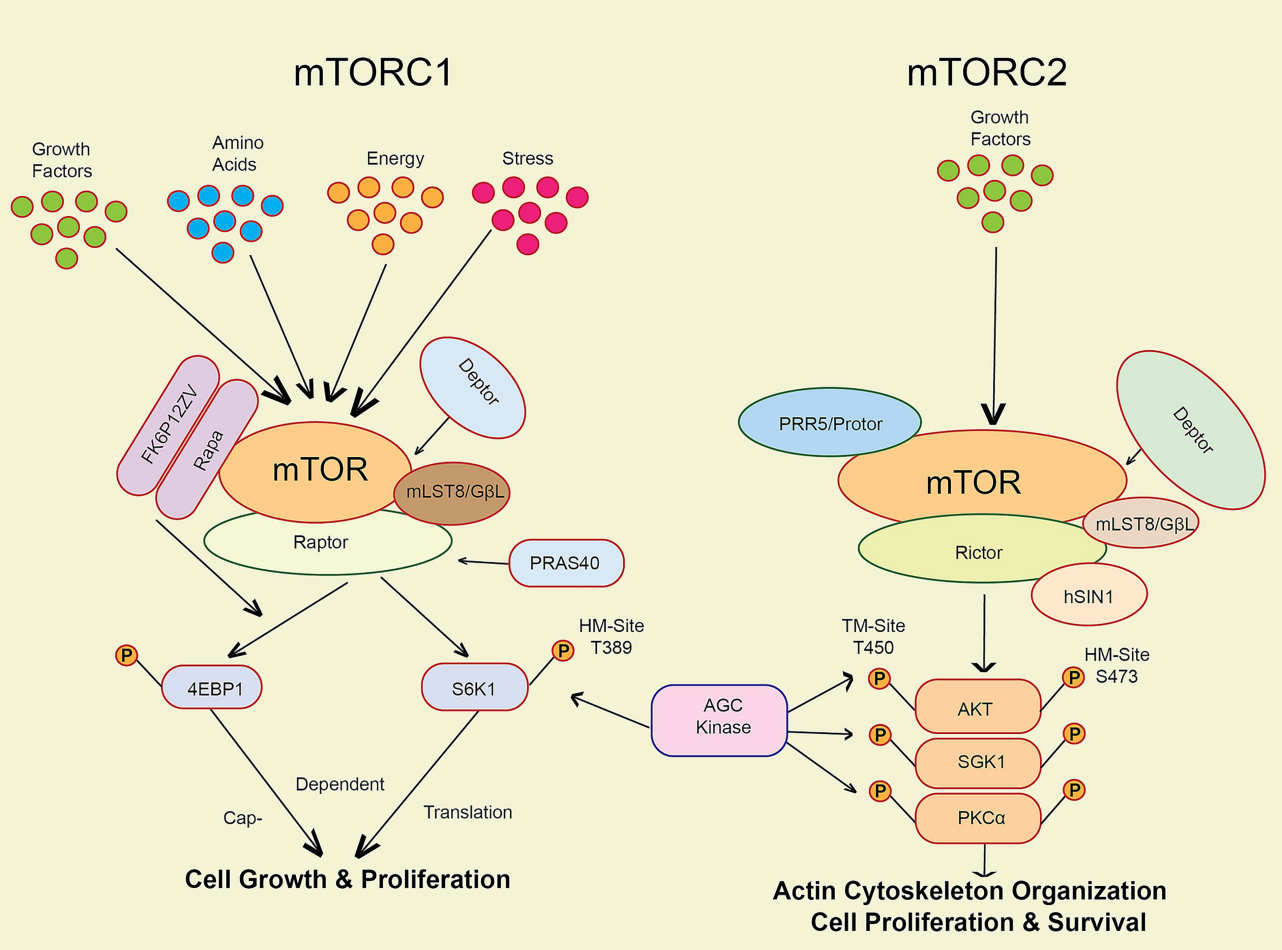
The key markers involved in the mTOR pathway activation via various factors like stress, energy, growth factors, amino acids and cellular senescence.

One of the most important cellular growth and metabolic regulators is the mTOR pathway (Zhu et al., 2019). The mTOR protein kinase is an essential part of two diverse complexes of protein known as mTORC1 and mTORC2, which are essential in the regulation of proliferation and survival of cells (Figure 1), and metabolism (Zhu et al., 2019; Chrienova et al., 2021).

mTOR regulates autophagy, a cellular degradation process essential for clearing misfolded proteins and damaged organelles. In AD, hyperactivation of mTOR leads to impaired autophagic flux, contributing to the accumulation of *β*-amyloid plaques and tau tangles, which are hallmarks of the disease (Subramanian et al., 2022; Querfurth and Lee, 2021). For instance, rapamycin, an mTOR inhibitor, has shown promise in promoting autophagy and reducing tau pathology in preclinical models, thereby potentially slowing cognitive decline (Zhu et al., 2019; Querfurth and Lee, 2021; Sarkar et al., 2009).

In PD, mTOR’s role is similarly significant. The accumulation of *α*-synuclein aggregates is linked to dysfunctional autophagy. Studies indicate that inhibiting mTOR can enhance the clearance of these aggregates, suggesting a therapeutic avenue for intervention (Zhu et al., 2019; Sarkar, 2013). Furthermore, recent research highlights that modulating the mTOR pathway can restore autophagic activity and reduce neurodegeneration across various models, reinforcing the potential for targeted therapies that balance mTOR signaling to enhance autophagic processes (Zhu et al., 2019). Understanding these pathways offers valuable insights into developing effective treatments for neurodegenerative conditions.

The figure illustrates the mTOR pathway, which regulates cell growth and metabolism through two complexes: mTORC1, activated by growth factors, stress, energy levels, and amino acids to promote protein synthesis, and mTORC2, activated by insulin and other growth factors to support cell survival. Both complexes help cells adapt to their environment and maintain proper function (Figure 1). Activation of mTORC1 through phosphorylation of eukaryotic initiation factor 4E binding protein 1 (4E-BP1), and the 70-kDa ribosomal protein S6 kinase (S6K1) can potentially increase protein synthesis while concurrently inhibiting autophagy (Chrienova et al., 2021; Perluigi et al., 2015). mTORC1 activation is initiated by growth hormones and amino acids (Perluigi et al., 2015). It governs the survival of cells and cytoskeletal architecture via the phosphorylation of AKT and other substrates. mTORC2 is activated when cells are exposed to growth factors like insulin (Zhu et al., 2019). Dysfunction in the mTOR pathway has been linked to various human diseases, such as cancer, metabolic disorders, and neurodegenerative conditions (McHugh and Gil, 2018; Perluigi et al., 2015). Evidence shows that mTOR activation plays a role in the development of harmful protein aggregates and the initiation of neuroinflammatory responses in neurodegenerative disorders (Maiese, 2016).

Several pharmaceuticals, such as rapamycin and its analogues, which block mTORC1 activity in a particular manner, have been developed to target the mTOR pathway (Chinta et al., 2018; Maiese, 2016). In preclinical models of Alzheimer’s disease, these pharmaceuticals have shown a high level of potential for treatment (Chinta et al., 2018; Zhu et al., 2019). Nevertheless, their use in clinical settings is restricted due to the possibility of adverse consequences, such as immunosuppression and metabolic dysregulation (Zhu et al., 2019).

#### Autophagy

**Figure 2:**
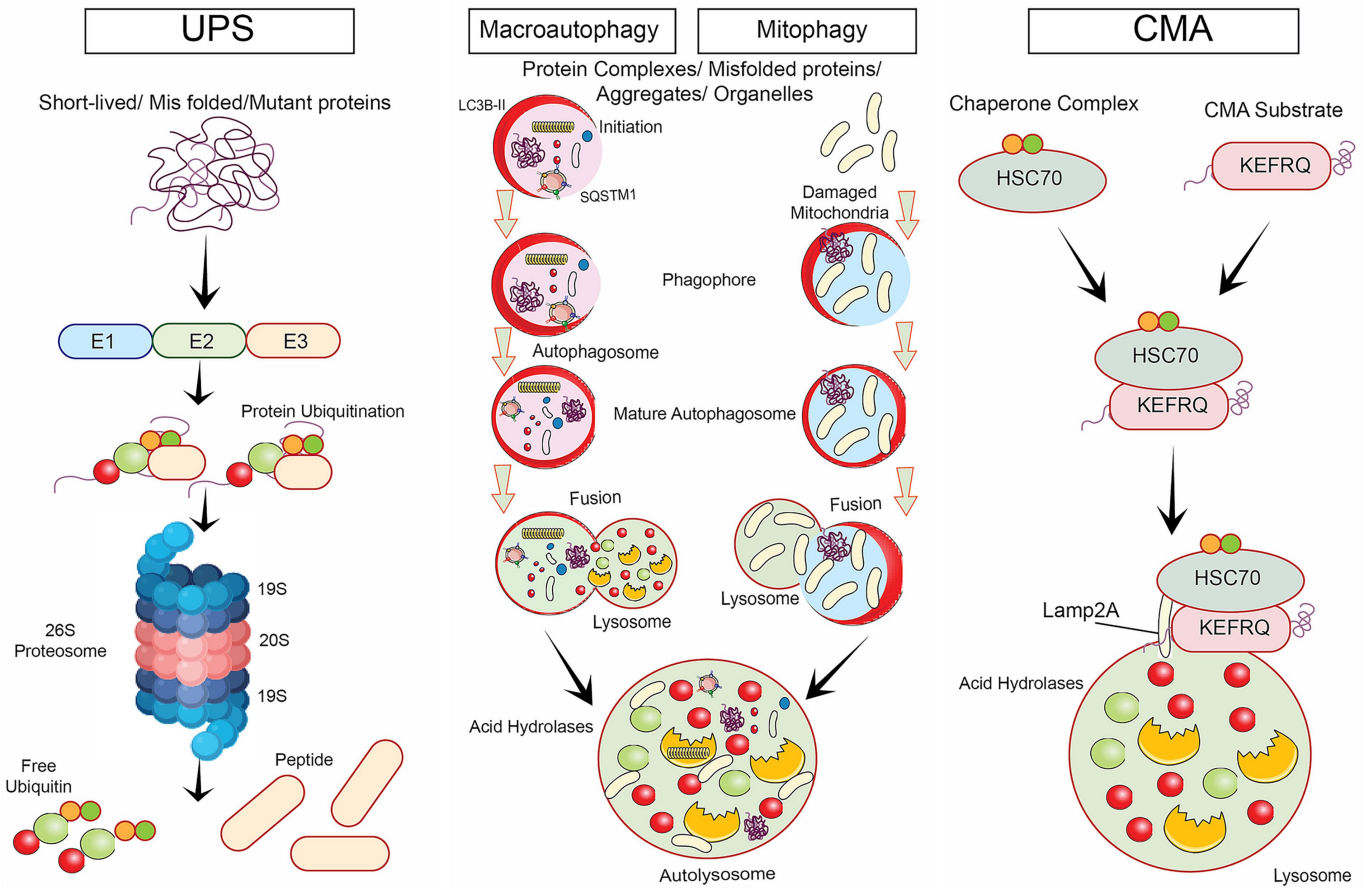
Various degradative signalling pathways involved in neurodegenerative diseases namely, ubiquitin proteasomal degradative mechanism, selective autophagy such as macro autophagy, mitophagy and chaperone-mediated autophagy.

Autophagy is a cellular mechanism accountable for breaking down and recycling of numerous cytoplasmic components, such as impaired organelles, protein aggregates, and intracellular pathogens, via the lysosomal pathway (Duan and Tong, 2021; Yim and Mizushima, 2021). It plays a key role in maintaining cellular homeostasis (Figure 2), and it has been linked to several healthy and unhealthy processes, such as cell ageing and neurodegeneration (Iyaswamy et al., 2022; Yang et al., 2017).

Various degradative signaling pathways involved in neurodegenerative diseases were illustrated. Key mechanisms include the UPS pathway, which targets misfolded proteins for destruction, and selective autophagy, which encompasses several processes: Macroautophagy, responsible for degrading large cellular structures; Mitophagy, which specifically removes dysfunctional mitochondria; and Chaperone-mediated autophagy, which transports specific proteins into lysosomes for degradation. Together, these pathways help maintain cellular homeostasis by preventing the accumulation of toxic proteins and damaged organelles.

The class III phosphatidylinositol 3-kinase (PI3K) complex, the Unc-51-like autophagy activating kinase 1 (ULK1) complex, and the autophagy-related gene (ATG) proteins are all components of the complex signaling network that regulates autophagy (Kageyama et al., 2021). mTORC1 is activated when nutrients are abundant (Perluigi et al., 2015). This inhibits autophagy by phosphorylating ULK1 and other targets later (Perluigi et al., 2015; Laplante and Sabatini, 2012). In contrast, mTORC1 is disabled when a cell lacks sufficient food. As a result, ULK1 is activated, and the process of autophagy is initiated (Kageyama et al., 2021; Laplante and Sabatini, 2012).

Neurodegenerative illnesses have been associated with the dysregulation of autophagy (Leidal et al., 2018). Recent research indicates that neurodegenerative diseases could be treated by manipulating autophagy (Caccamo et al., 2013; Iyaswamy et al., 2021; Zhu et al., 2022). Administering drugs like rapamycin to enhance autophagy has shown the potential to delay the onset of neurodegeneration in animal models of AD and PD (Spilman et al., 2010; Decressac et al., 2012; Iyaswamy et al., 2024). In few investigations, certain small molecules that activate autophagy, such as trehalose and lithium, have exhibited potential as prospective therapeutics for conditions leading to neuronal cell death (Dossymbekova et al., 2020; Rusmini et al., 2019; Pandaram et al., 2024).

#### Lysosomal biogenesis

Lysosomes are cellular organelles that destroy pathogens, macromolecules, and damaged organelles through the process of autophagy (Sreenivasmurthy et al., 2022; Tong et al., 2022). They also degrade other cellular components including defective organelles (Wang et al., 2021; Guan et al., 2022). The process of creating new lysosomes, known as lysosomal biogenesis, is controlled by a complex interplay of signaling pathways, including the mTOR pathway and transcription factors like Transcription factor EB (TFEB) and transcription factor E3 (TFE3) (Iyaswamy et al., 2022; Wang et al., 2020; Song et al., 2020).

TFEB plays a crucial role in regulating both lysosomal biogenesis and autophagy (Leidal et al., 2018; Song et al., 2020). Phosphorylation and the transport of proteins between the nucleus and cytoplasm regulate its activity (Wang et al., 2020). mTOR phosphorylates TFEB, which retains it in the cytoplasm and shuts it off when many nutrients present in the cell (Leidal et al., 2018; Song et al., 2020). TFEB loses its phosphorylation and goes into the nucleus, activating genes associated with lysosomes and autophagy (Table 2], when there are insufficient nutrients, such as under starvation or lysosomal stress (Settembre et al., 2011; Martina et al., 2014). This promotes autophagy and lysosome biogenesis (Settembre et al., 2011; Martina et al., 2014).

**Table 2:** Key regulators of autophagy and lysosomal biogenesis, indicating their specific function and regulating selective autophagy, UPS, and other degradative mechanisms.

Pathway	Definition	Key regulators	Role
mTOR (Laplante and Sabatini, 2012; Caccamo et al., 2013)	A signalling cascade that governs cell growth and metabolic processes in reaction to the presence of nutrients and growth factors.	mTORC1, mTORC2, AKT, AMPK, TSC1/2, Rheb	Promotes protein synthesis, cell growth, and inhibits autophagy in nutrient-rich conditions.
Autophagy (Boland et al., 2008)	A catabolic mechanism characterized by the breakdown and repurposing of cellular elements, including impaired organelles and proteins, to uphold cellular equilibrium.	ULK1, Beclin1, VPS34, LC3, p62	Removes damaged organelles and proteins and provides energy during nutrient deprivation.
Lysosomal Biogenesis (Medina et al., 2015; Ciechanover and Kwon, 2015)	A process that involves the formation and maturation of lysosomes, which are responsible for the degradation of intracellular material delivered by autophagy.	TFEB, TFE3, MiT/TFE, RAB7, LAMP1/2	Regulates lysosomal biogenesis, acidification, and function.
UPS (Keller et al., 2000; Sweeney et al., 2017)	A proteolytic mechanism responsible for breaking down cytosolic and nuclear proteins through the action of the proteasome.	E1, E2, E3 ubiquitin ligases, 26S proteasome	Regulates protein quality control, antigen processing, and cell signaling.
Mitophagy (Palikaras et al., 2018; Liu et al., 2012)	A distinctive form of autophagy that includes the breakdown of impaired or surplus mitochondria to ensure cellular homeostasis.	PINK1, Parkin, FUNDC1, BNIP3	Regulates mitochondrial quality control

Other transcription factors have also been connected to regulating the advancement and action of lysosomes, including TFE3, microphthalmia-associated transcription factor (MITF), and zinc finger protein with KRAB and SCAN domains 3 (ZKSCAN3) (Sardiello et al., 2009; Palmieri et al., 2011; Medina et al., 2015). The formation of lysosomes is also governed by post-transcriptional mechanisms, such as mRNA processing, stability, and translation that regulate the expression of lysosomal proteins (Medina et al., 2015). Overall, issues with lysosomal biogenesis have been connected to several diseases, including lysosomal storage disorders and neurodegenerative diseases (Medina et al., 2011; Boland et al., 2008; Torra et al., 2018). Lysosomal biogenesis is a crucial component of maintaining cellular homeostasis (Boland et al., 2008).

### Triggers

**Figure 3:**
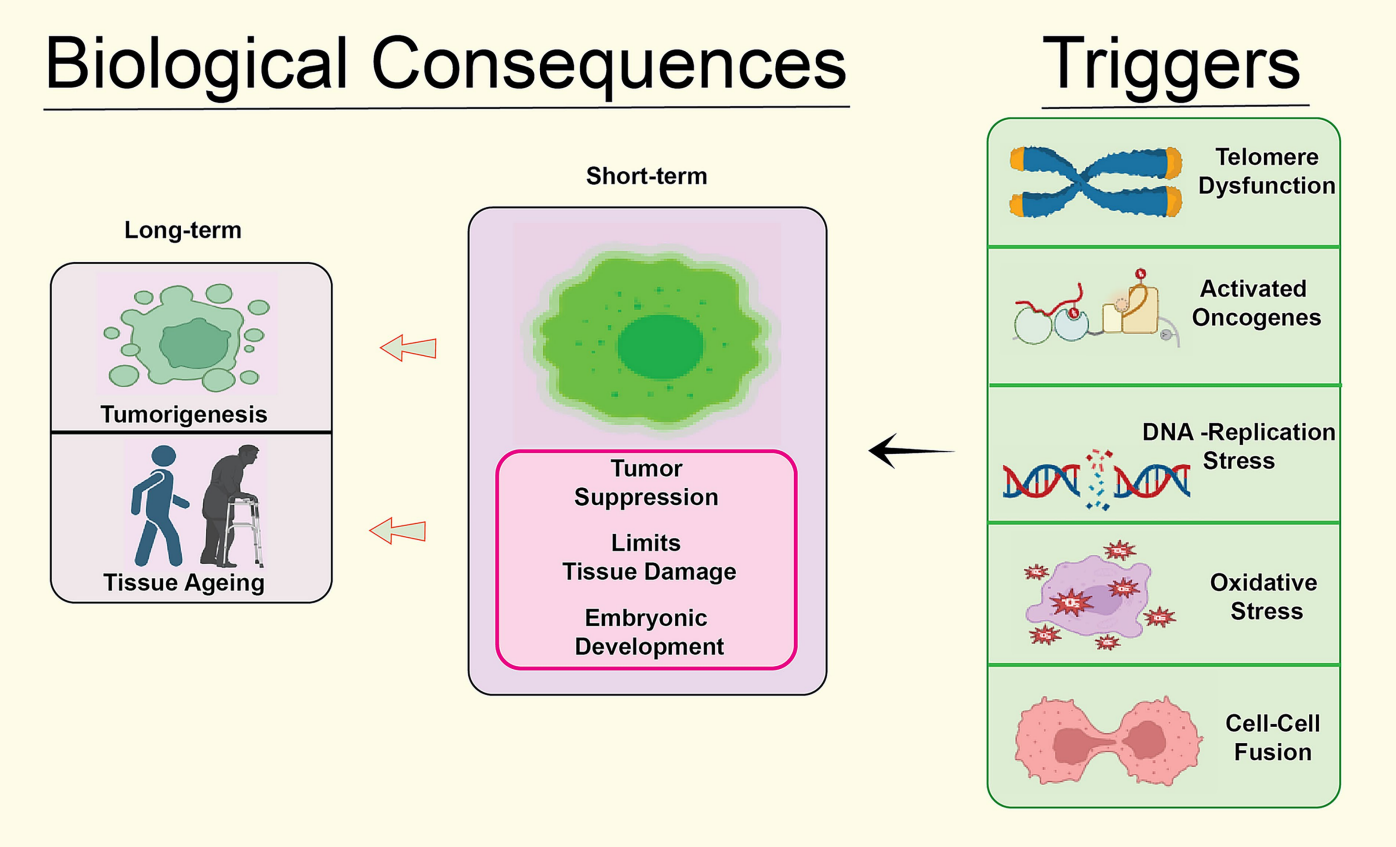
Biological consequences of the cellular senescence caused by different risk factors such as oxidative stress, telomere dysfunction, replication mistakes, DNA damage and stress in different forms.

Cellular senescence represents a permanent arrest of cell cycle, which may be produced by a category of events, both intrinsic and extrinsic. DNA damage, shortening of telomeres, replication stress, and ROS [Figure 3) are the four basic categories that may be used to classify the triggers (Lopez-Otin et al., 2013).

A process known as DNA damage-induced senescence (DIS) is set in motion when double-stranded DNA breaks (DSBs) or other forms of DNA damage occur (Campisi, 2005). The presence of double-strand breaks triggers the DNA damage response (DDR), which then activates the ataxia-telangiectasia mutated (ATM) and Rad3-related (ATR) kinases (Campisi, 2005). The activation of these kinases results in a halt in the cell cycle and the senescence initiation, leading to the phosphorylation of p53 and other downstream effectors. Increasing evidence suggests that DIS plays a pivotal role in the progression of various age-related diseases, including cancer and neurodegenerative diseases (Childs et al., 2015).

Telomere shortening, a process characterized by the continuous reduction in the length of repeating DNA sequences located at the chromosome ends, is responsible for the occurrence of telomere shortening-induced senescence (TSIS) (Babizhayev and Yegorov, 2015). The normal process of telomere shortening happens whenever a cell divides; however, this natural process may be balanced out by the action of telomerase (Babizhayev and Yegorov, 2015; Shay, 2018). Telomeres naturally become shorter when cells divide (Shay, 2018). Eventually, the telomeres of cells that do not produce telomerase, such as somatic cells, become dangerously short, resulting in activation of DNA damage response and senescence (Razgonova et al., 2020; Lansdorp, 2022). There is evidence that TSIS has a role in both ageing and the illnesses that are associated with aging (Razgonova et al., 2020; Lansdorp, 2022).

The buildup of stalled or collapsed replication forks is what causes replication stress-induced senescence (RSIS), which may be caused by several different circumstances, including replication mistakes, DNA damage, and nucleotide depletion. The activation of DDR pathways in response to replication stress leads to the inhibition of the cell cycle and the initiation of senescence (Pedroza-Garcia et al., 2022). The RSIS gene has been demonstrated to play a role in the development of age-related illnesses and cancer pathogenesis (Pedroza-Garcia et al., 2022; Dhama et al., 2019).

The buildup of ROS, which are byproducts of cellular metabolism, is the fundamental trigger for ROS-induced senescence (ROSIS), also known as senescence induced by ROS (Fakouri et al., 2019). The oxidative injury inflicted by ROS on cellular elements such as DNA, proteins, and lipids can ultimately initiate the activation of DDR and senescence pathways (Fakouri et al., 2019; Reddy et al., 2017). ROSIS has been demonstrated to actively contribute to the etiology of aging and age-related disorders, such as neurodegenerative diseases (Reddy et al., 2017).

**Figure 4:**
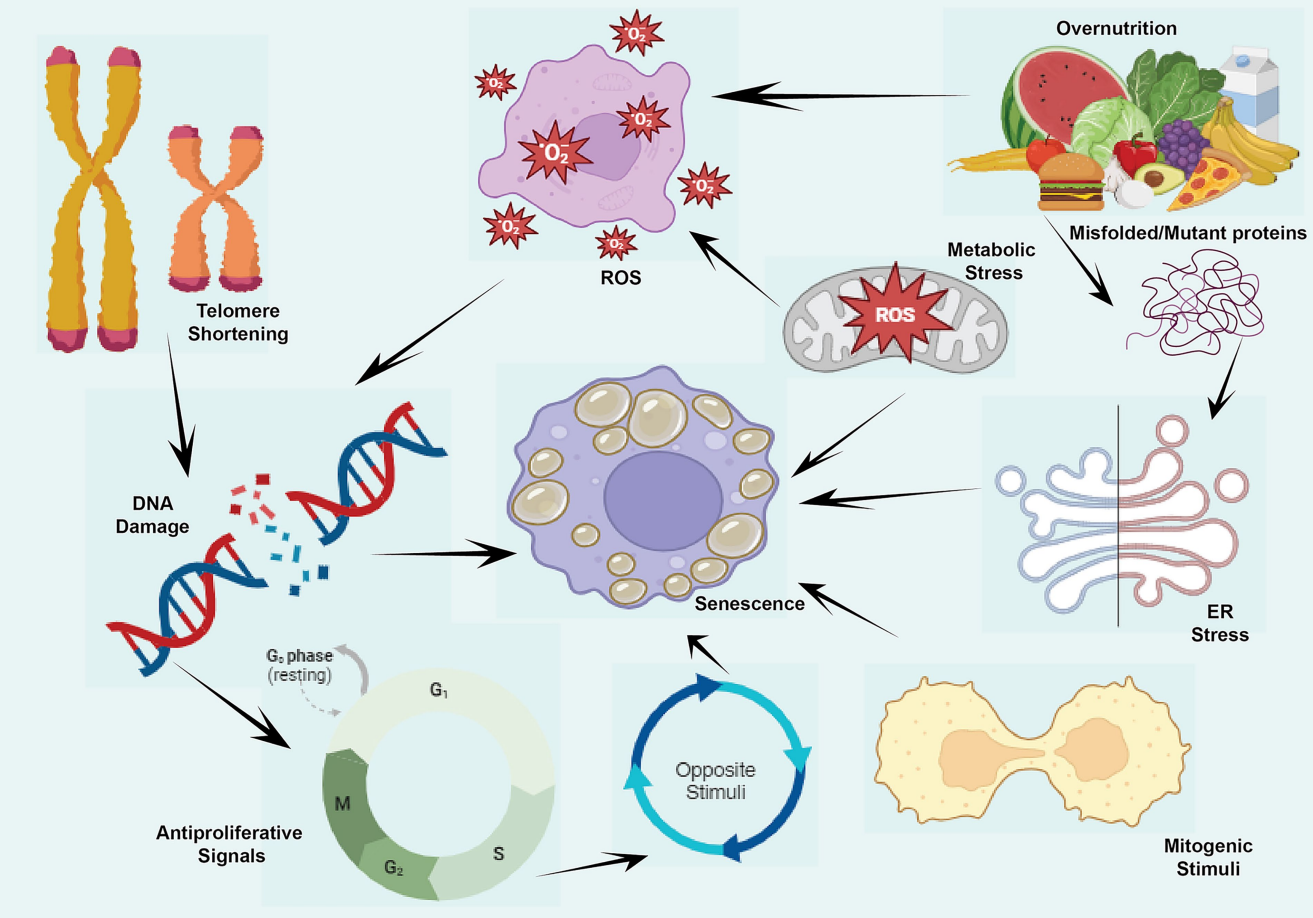
Cellular senescence and it causes or stimuli namely antiproliferative signals, DNA damage, ER stress, misfolded proteins, metabolic stress, ROS production and mitogenic stimuli during the aging.

In summary, cellular aging can be initiated by a diverse array of internal and external factors, involving DNA damage, shortened telomeres, replication stress, and ROS (Reddy et al., 2017). It is necessary to have a solid understanding of the causes of senescence to be able to devise methods that may postpone or prevent illnesses associated with aging [Figure 4).

This figure illustrates the various stimuli and consequences of cellular senescence. Triggers such as DNA damage occurs due to events like double-stranded DNA breaks (DSBs), which activate the DNA damage response (DDR) and lead to the phosphorylation of p53 and other downstream effectors, initiating DNA damage-induced senescence (DIS). Telomere Shortening results from repeated cell divisions, leading to critically short telomeres that activate DDR and promote telomere shortening-induced senescence (TSIS). Replication Stress arises from stalled replication forks caused by replication errors, DNA damage, or nucleotide depletion, resulting in replication stress-induced senescence (RSIS) through DDR activation and cell cycle inhibition. Lastly, Reactive Oxygen Species (ROS), byproducts of cellular metabolism, induce oxidative damage to DNA, proteins, and lipids, triggering ROS-induced senescence (ROSIS) by activating DDR pathways. To sum up, these senescence pathways contribute to the progression of age-related diseases, including cancer and neurodegenerative disorders.

### Senescent cells in neurodegenerative diseases

Several neurodegenerative disorders have been linked to the existence of senescent cells. Senescent cells are observed in neurons, microglia, and astrocytes of individuals with AD (Reddy et al., 2017; Slanzi et al., 2020). It has been reported that senescent astrocytes are a contributing factor in amyloid-beta plaques production in the brain (Reddy et al., 2017).

### Alzheimer’s disease (AD)

AD is a neurodegenerative condition described by the buildup of beta-amyloid plaques and neurofibrillary tangles within the brain (Reddy et al., 2017). This accumulation eventually causes the death of neurons and cognitive decline. The progression of AD has been linked to the senescence process observed in neurons, microglia, and astrocytes (Swerdlow and Khan, 2004).

p16 and p21, the senescence markers have been detected in the brains of individuals diagnosed with Alzheimer’s disease (Swerdlow and Khan, 2004). These markers related to tau pathology and neuronal loss are found in neurons (Bussian et al., 2018; Heneka et al., 2013). In addition, an accumulation of damaged DNA and oxidative stress may promote senescence in neurons, which results in a decline in mitochondrial function, synaptic dysfunction, and upregulated production of pro-inflammatory cytokines (Selvarasu et al., 2023). Senescence can also cause the death of neurons (Bussian et al., 2018; Heneka et al., 2013). When the brain is injured or infected, the resident immune cells known as microglia play a key role in both the maintenance of homeostasis and the response to the condition (Bussian et al., 2018; Nicaise et al., 2019). Microglia become persistently activated and create a proinflammatory phenotype in AD, which contributes to the neuroinflammation seen in this condition (Nicaise et al., 2019). Senescence in microglia has been connected to SASP factors development, which may make neuroinflammation and neurodegeneration worse (Nicaise et al., 2019; Freund et al., 2010).

Astrocytes, the predominant form of glial cells in the brain, offer structural and metabolic support to neurons (Freund et al., 2010). Evidence of enhanced expression of p16 and p21 in the brains of AD patients suggests that senescence in astrocytes may perform a vital role in the development of the disease (Sahu et al., 2022; Li et al., 2021). The ageing of astrocytes may result in a reduction in neurotrophic support, an impairment of glucose metabolism, and upregulated inflammation (Sahu et al., 2022; Regen et al., 2017). Some evidence suggests that blocking senescence in neurons, microglia, and astrocytes could be an effective therapy for AD (Sahu et al., 2022; Regen et al., 2017). Studies have shown that senolytic drugs, targeting senescent cells selectively, hold promise in enhancing cognitive abilities and mitigating neuroinflammation in mouse models of AD (Regen et al., 2017; Beraud and Maguire-Zeiss, 2012; Kang, 2019).

Parkinson’s Disease (PD) impacts dopaminergic neurons located in the substantia nigra region of the brain, which deteriorates over time (Beraud and Maguire-Zeiss, 2012). Many investigations have led researchers to the conclusion that cellular senescence is a critical factor in the development of PD (Beraud and Maguire-Zeiss, 2012).

Markers of senescence-like p16, p21, and p53 present in dopaminergic neurons, astrocytes, and microglia in the substantia nigra are affected by PD (Tian et al., 2021; Hohn et al., 2017). In addition, DNA damage indicators, including gamma-H2AX and 8-hydroxydeoxyguanosine (8-OHdG) were discovered in these cells (Hohn et al., 2017). According to these indicators, DNA damage-induced senescence could perform a vital role in the development of PD (Kang, 2019; Hohn et al., 2017). In addition, previous research has shown that oxidative stress caused by dysfunction of mitochondrial may bring about cellular senescence in PD patients (Kang, 2019; Schrauwen et al., 2010). Senescence can result from mitochondrial dysfunction, along with oxidative stress, which is correlated with the buildup of damaged proteins, impaired autophagy, and compromised mitochondrial quality control (Hohn et al., 2017; Schrauwen et al., 2010).

In summary, cellular aging, initiated by DNA damage and oxidative stress, could potentially play a role in the development of PD by instigating senescence in dopaminergic neurons, astrocytes, and microglia (Hohn et al., 2017). This may be the case because cellular senescence causes dopaminergic neurons to die off (Hohn et al., 2017). Advancing the discovery of markers for senescence and uncovering the underlying processes in PD could enhance the identification of novel therapeutic targets for treating the condition (Kang, 2019).

### Amyotrophic lateral sclerosis (ALS)

ALS, a neurodegenerative condition, is characterized by the gradual degeneration of motor neurons in both the brain and spinal cord (Pandya and Patani, 2020). The process of cellular senescence has been linked to the onset of ALS (Pandya and Patani, 2020). Postmortem examination of the spinal cords of ALS patients has shown higher levels of senescence markers such as p16 and p21 in the motor neurons, astrocytes, and microglia of these tissues (Ziff and Patani, 2019). Studies in individuals with ALS have demonstrated that aging cells display a pro-inflammatory SASP, contributing to the advancement of the disease (Oh et al., 2016). The SASP is distinguished by increased production and release of pro-inflammatory cytokines, chemokines, and matrix metalloproteinases in individuals with ALS, potentially resulting in chronic inflammation and tissue damage if not addressed (Pandya and Patani, 2020; Oh et al., 2016).

In ALS, senescent cells exhibit compromised autophagy and lysosomal function, which may result in the aggregation of dysfunctional organelles and protein aggregates (Pandya and Patani, 2020). This is in addition to SASP, a known risk factor for the disease. This buildup may make inflammation and neurodegeneration in ALS patients even worse (Liddelow and Barres, 2017). In the treatment of ALS, one possible therapeutic technique is to target cellular senescence. Recent studies demonstrated that treatment utilizing senolytics, compounds designed to induce apoptosis specifically in senescent cells, improved motor function and prolonged lifespan in a mouse model of ALS (Maximova et al., 2021). Considering the evidence linking senescent cells to the development of ALS, targeting these cells in treatment could offer a promising therapeutic approach for this debilitating illness (Maximova et al., 2021).

### Huntington disease (HD)

The neurodegenerative condition known as HD is passed down through families and is brought on by a change in the huntingtin gene (Jiang et al., 2011). The gradual degeneration of the striatum, which is the part of the brain that is important for both motor control and cognition, is the defining characteristic of HD (Jiang et al., 2011; Cohen and Torres, 2019). The concept of cellular senescence has been proposed as a potential factor in the onset of HD. Several studies have revealed indications of the existence of senescent cells in the brains of individuals with HD (Jiang et al., 2011; Cohen and Torres, 2019). Based on previous research, individuals with HD exhibit a buildup of senescent astrocytes in their brains, potentially resulting in neuroinflammation and the subsequent death of neurons in the neighboring regions (Liddelow and Barres, 2017; Cohen and Torres, 2019). Another research in HD animal models found senescent microglia in the striatum, which may be a factor in the progression of neuroinflammation and synaptic dysfunction (Liddelow and Barres, 2017; Jiang et al., 2011; Cohen and Torres, 2019).

It is possible that several other factors such as oxidative stress, damage to DNA, and shortening of telomeres, are to blame for the increase of senescent cells in HD (Acosta et al., 2013). Moreover, the mutant huntingtin protein can directly promote cellular senescence by activating the p53 pathway (Tian et al., 2021). As a potential treatment for HD, one strategy that shows promise targets cellular senescence (Tian et al., 2021; Jiang et al., 2011). Research findings demonstrated that inhibiting the p53 pathway enhanced motor function in a rat model of HD by lowering the number of senescent astrocytes that accumulated in the brain (Tian et al., 2021; Jiang et al., 2011; Cohen and Torres, 2019). In summary, it appears that cellular aging is significantly associated in the development of HD. Targeting senescent cells could emerge as a promising therapeutic approach for addressing this severe neurodegenerative condition (Jiang et al., 2011).

### Senolytics

Senolytics are a group of drugs or plant chemicals that target and kill senescent cells in a specific way (Kang, 2019; Xu et al., 2018). In recent years, these compounds have gained a lot of attention because they might be able to treat age-related diseases (Kang, 2019; Xu et al., 2018; Zhu et al., 2015).

Senolytic drugs target specific molecular markers of cellular senescence, primarily p16Ink4a and p21Cip1/Waf1, which play crucial roles in neurodegenerative conditions. Senescence is characterized by cell cycle arrest mediated by these markers, with p21 acting downstream of the p53 pathway and p16 upstream of the RB pathway. This interaction leads to irreversible cell cycle arrest and contributes to the SASP, which exacerbates neurodegenerative diseases by promoting inflammation and tissue dysfunction (Kudlova et al., 2022; Wagner and Wagner, 2022; Chaib et al., 2022). Different senolytic agents, such as dasatinib and quercetin (D + Q), selectively eliminate senescent cells expressing high levels of p16 or p21. Their efficacy varies across neurodegenerative conditions due to the heterogeneous nature of senescent cells influenced by various stressors like oxidative damage or DNA damage responses (Chaib et al., 2022; Gasek et al., 2021). For instance, in AD models, targeting p21 may alleviate cognitive decline by reducing SASP factors that promote neuroinflammation (Kumari and Jat, 2021; Fan et al., 2024). Apparently, in models of PD, p16-targeting strategies may be more effective due to distinct cellular responses to stressors (Wagner and Wagner, 2022; Gasek et al., 2021). Thus, understanding these interactions is vital for optimizing senolytic therapies tailored to specific neurodegenerative conditions.

**Figure 5:**
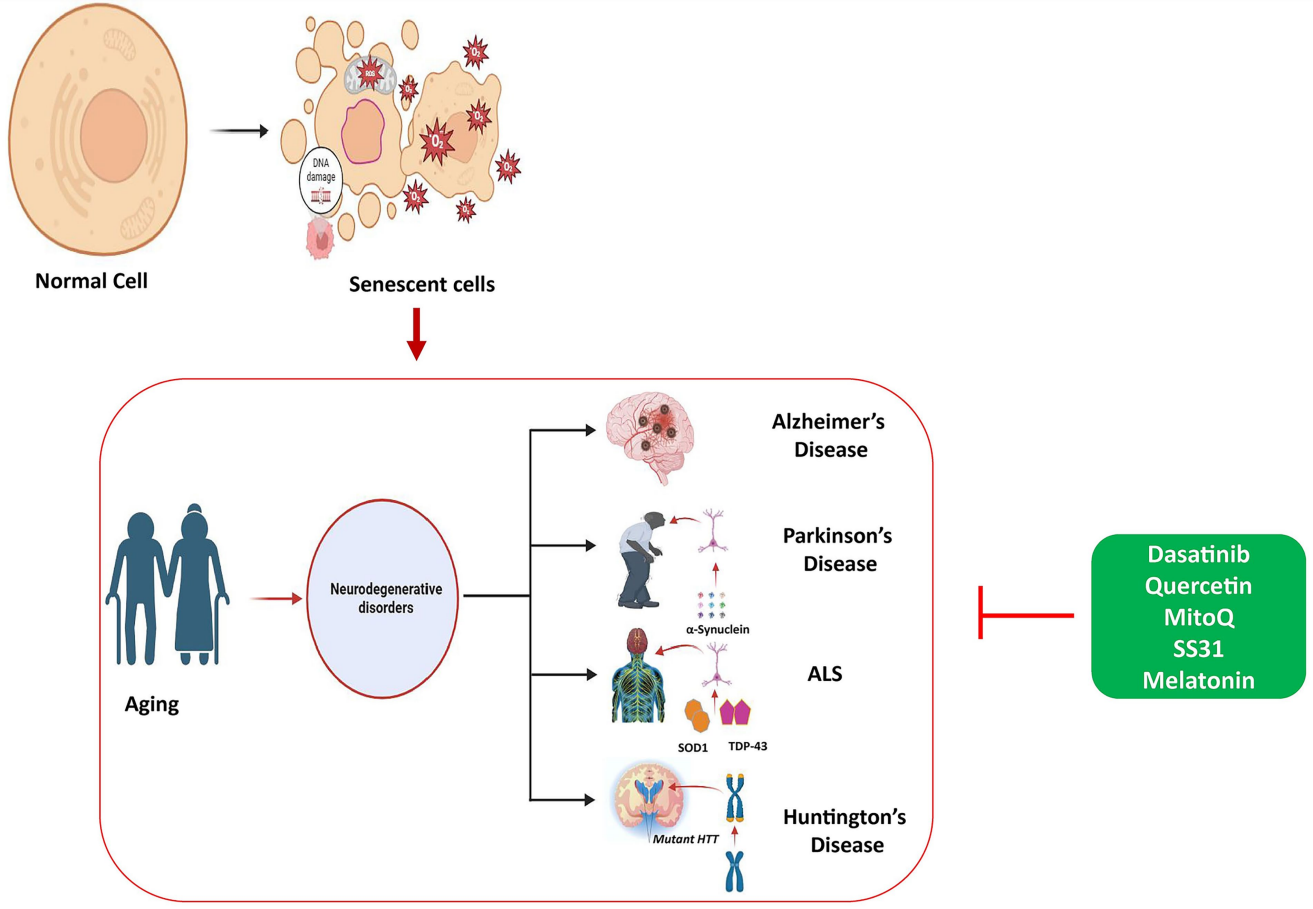
Role of cellular senescence in aging and accumulation of senescent cells causing aging pathology. Therapeutic strategies using different types of senolytics including phytochemicals and antioxidant medications, alleviate the pathogenesis of for neurodegenerative diseases, targeting key molecular pathways.

Dasatinib and quercetin form a well-established senolytic combination proven to eliminate aged cells across various tissues, resulting in improved functionality in aging mice (Kang, 2019; Zhu et al., 2015). In preclinical studies, other senolytic compounds [Figure 5), such as fisetin, have also shown promise (Zhu et al., 2015; Kirkland and Tchkonia, 2020). These substances function by inducing the death of senescent cells through various mechanisms, including the inhibition of anti-apoptotic pathways and promoting the initiation of apoptosis in senescent cells (Kirkland and Tchkonia, 2020). Senolytics show a lot of promise to treat diseases related to aging by stopping or reversing cellular senescence (Xu et al., 2018; Zhu et al., 2015).

Cellular senescence contributes to aging as senescent cells accumulate due to increased survival signals and reduced cell death, leading to tissue damage caused by inflammatory SASP factors. Treatments include senolytics, which remove these harmful cells, and senomorphics, reduce their damaging secretions, with effects on ageing and neurodegenerative diseases.

This figure illustrates the role of cellular senescence in aging, showing how the accumulation of senescent cells contributes to age-related diseases. Cellular senescence leads to reduced tissue function and the progression of aging-related pathology. The figure also outlines therapeutic strategies using senolytics and senomorphics that aim to enhance healthspan and reduce the effects of cellular senescence on aging and neurodegenerative diseases.

### Types of senolytics

- Dasatinib and Quercetin: Dasatinib is a tyrosine kinase inhibitor, while quercetin is a flavonoid molecule found in a wide range of fruits and vegetables.
- Studies have suggested that using these two medications simultaneously might selectively eliminate aged cells by inhibiting the survival pathways utilized by those cells (Zhu et al., 2015; Novais et al., 2021; Saccon et al., 2021).
- The flavonoid fisetin is commonly present in a diverse range of fruits and vegetables, including strawberries, apples, and persimmons. Fisetin is a compound that occurs naturally. It has been demonstrated to specifically trigger apoptosis in aging cells (Zhu et al., 2017; Yousefzadeh et al., 2018).
- Navitoclax molecule is a tiny chemical that inhibits anti-apoptotic proteins Bcl-2 and Bcl-xL. Research indicates that selectively inducing apoptosis in senescent cells can be achieved by blocking their anti-apoptotic pathways (Zhu et al., 2017; Zhu et al., 2016).
- Selisistat is a selective inhibitor of the histone deacetylase (HDAC) enzyme. Selisistat is also known as selesistance. It has been shown that by blocking the pro-survival pathways of senescent cells, may specifically trigger apoptosis in those cells (Sikora et al., 2010; Smith et al., 2014).
- ABT-263 is a tiny chemical discovered accidentally that inhibits Bcl-2 and Bcl-xL. Research has demonstrated that inhibiting the anti-apoptotic pathways of senescent cells could selectively induce apoptosis in those cells (Zhang et al., 2021; Tse et al., 2008).
- UBX0101: UBX0101 is a small chemical compound that functions as an inhibitor, interrupting the interaction between MDM2 and p53. It has been shown that activating the p53 pathway, may trigger apoptosis in senescent cells in a selective manner (Kirkland and Tchkonia, 2020; Wang et al., 2017; Zafar et al., 2021).

### Role of Senolytics in neurodegenerative diseases

Neurodegenerative diseases are characterized by a gradual deterioration in neuronal function, leading to eventual cell death, resulting in impaired cognitive and motor processes (Regen et al., 2017). With aging, there is a buildup of senescent cells that release pro-inflammatory and toxic substances, potentially leading to impaired neuronal function and the promotion of neuroinflammation (Baker et al., 2011; Freund et al., 2010; Regen et al., 2017).

The buildup of senescent cells has been recognized as a potential contributor to the onset of neurodegenerative diseases, underscoring the involvement of cellular senescence in their development (Baker et al., 2011; Freund et al., 2010). In preclinical investigations, usage of senolytics, has shown significant promise as a possible therapy for neurodegenerative illnesses (Maximova et al., 2021; Cohen and Torres, 2019).

In preclinical models of AD and PD, senolytics (Table 3) have been confirmed to be efficient in several trials in improving cognitive and motor impairments (Baker et al., 2011; Bussian et al., 2018; Maximova et al., 2021; Xu et al., 2018). Dasatinib, a senolytic medication, plus quercetin, an antioxidant, enhanced cognitive function in a mouse model of AD by lowering the number of senescent cells present in the brain (Baker et al., 2011; Bussian et al., 2018; Xu et al., 2018). Senolytic medication ABT-263 enhanced motor function and decreased the growth of aging cells in a mouse model of PD (Zhu et al., 2017; Tse et al., 2008). Similarly, ALS mouse model administered with senolytic medication navitoclax resulted in an enhancement in motor function and a reduction in neuroinflammation (Zhu et al., 2017; Liu et al., 2022).

**Table 3:** Therapeutic strategies using different types of senolytics including phytochemicals and antioxidant medications, alleviates the pathogenesis of neurodegenerative diseases and its potential targets.

Class / type	Example compounds	Primary Targets / Pathways	Detailed mechanism of action	Neurodegenerative diseases	Senescence-related impact
Senolytic combo	Dasatinib + Quercetin (D + Q)	Src family kinases (dasatinib); PI3K/NF-κB, ROS (quercetin)	Disables SCAP (senescent cell anti-apoptotic pathways), promotes apoptosis selectively in senescent cells	AD, PD, age-related cognitive decline	Reduces SASP cytokines (IL-6, IL-1β); mitigates microglia/astrocyte-driven neuroinflammation
Flavonoid senolytic	Fisetin	PI3K/AKT, NF-κB, oxidative stress	Induces apoptosis in senescent cells, antioxidant effect	AD, PD	Lowers SASP; decreases glial activation
BCL-2 family inhibitors	Navitoclax (ABT-263), ABT-737( C42H45ClN6O5S2)	BCL-2, BCL-xL, BCL-w	Inhibits anti-apoptotic proteins, restores apoptosis in senescent cells	AD, PD (concept), ALS	Removes senescent glia; limits pro-inflammatory secretome
Peptide-based senolytic	FOXO4-DRI	FOXO4-p53 interaction	Releases p53 to trigger apoptosis in senescent cells	AD, PD (concept)	Targets p53/FOXO dysregulation in senescent neurons/glia
HSP90 inhibitors	17-DMAG, geldanamycin derivatives	HSP90 chaperone proteins	Destabilizes survival proteins in senescent cells	AD (inflammation-related)	Potential clearance of CNS senescent cells; cytotoxicity risk
p53 axis modulators	UBX0101, Nutlin-3	MDM2-p53-CDK	Local elimination of senescent cells	Joint senescence (models), concept in CNS	Strategy for targeted CNS senolysis
Natural senolytics	Piperlongumine, curcumin analogs	NF-κB, ROS	Promotes oxidative stress in senescent cells, inhibits SASP	AD, PD	Anti-inflammatory via SASP suppression
Targeted senolysis	Senescent cell surface-marker ADCs	Cell surface proteins on senescent cells	Precision drug delivery to induce apoptosis	AD, PD, ALS	CNS-selective clearance of senescent glia
Senomorphic (mTOR inhibitors)	Rapamycin, rapalogs	mTORC1, AKT, AMPK, TSC1/2	Inhibits mTORC1, suppresses SASP, enhances autophagy	AD, PD	Restores proteostasis, reduces Aβ/tau, limits SASP
Senomorphic (JAK inhibitors)	Ruxolitinib, Baricitinib	JAK1/2	Blocks IL-6/IL-8 SASP amplification	AD, ALS	Reduces systemic and CNS inflammaging
Senomorphic (p38 MAPK inhibitors)	SB203580(C21H16FN3OS), MW150	p38 MAPK	Attenuates NF-κB activation, reduces SASP cytokines	AD, PD, ALS	Lowers glial inflammatory output
Senomorphic (BET inhibitors)	JQ1, I-BET762	BRD4, epigenetic enhancers	Epigenetically silences SASP genes	AD (concept)	Broad SASP program suppression
Senomorphic (NF-κB modulation)	NF-κB inhibitors, dexamethasone	IKK/NF-κB	Suppress pro-inflammatory genes	AD, PD	Reduces microglial activation
Metabolic senomorphic	Metformin	AMPK, mTOR, NF-κB	Activates AMPK, suppresses SASP, enhances autophagy	AD, PD	Improves proteostasis, reduces Aβ/tau
Sirtuin activators	Resveratrol, SIRT1 activators	SIRT1	Inhibits NF-κB, promotes autophagy	AD, PD	Anti-inflammatory and proteostasis-supportive
Epigenetic SASP suppression	HDAC inhibitors	Histone acetylation	Alters chromatin to reduce inflammatory gene expression	AD (concept)	Suppresses SASP transcription
EV-based senomorphics	Exosomes from young MSCs, platelet-derived	EV cargo proteins/miRNAs	Reprograms senescent phenotype	AD, PD (preclinical)	Reduces senescence markers in glia
Autophagy enhancers	Rapamycin, metformin, spermidine	ULK1, Beclin1, VPS34, LC3, TFEB	Induce macroautophagy, lysosomal biogenesis, mitophagy	AD, PD	Clears Aβ, tau, α-syn, damaged mitochondria
Proteostasis/UPS modulators	MG132 analogs (concept)	E1/E2/E3 ligases, 26S proteasome	Restores UPS degradation	AD, PD	Prevents protein aggregation-driven senescence
Mitophagy inducers	Urolithin A, NAD + boosters	PINK1, Parkin, BNIP3, FUNDC1	Enhances mitochondrial quality control	PD, AD	Removes dysfunctional mitochondria, preventing SASP induction

MitoQ is a mitochondria-specific antioxidant that effectively inhibits oxidative damage to mitochondria (Wani et al., 2011). Recent study indicate that MitoQ can enhance mitochondrial activity, reduce oxidative stress-related aging, and lower reactive oxygen species (ROS) generation (Braakhuis et al., 2018). Using cellular models of prion disease, we examined MitoQ’s neuroprotective properties. Wu et al. (2025) demonstrate that MitoQ significantly reduces oxidative stress, mitochondrial dysfunction, and apoptosis caused by PrP106-126 by modifying DRP1- and OPA1-mediated mitochondrial dynamics. These results highlight MitoQ’s potential as a treatment for prion-induced neurodegeneration. In the 6-OHDA cell model of Parkinson’s disease, the mitochondria-targeted scavenger MitoQ decreases certain features of mitochondrial fission. Additionally, MitoQ prevented the pro-apoptotic protein Bax from moving to the mitochondria (Solesio et al., 2013). Furthermore, SS31 is a cell-permeable antioxidant peptide that targets mitochondria, reducing the formation of mitochondrial ROS, protecting mitochondrial structure, and alleviating mitochondrial dysfunction (Reddy et al., 2017). In APP/PS1 transgenic mice, increased levels of Aβ40/Aβ42 and the mitochondrial fission protein DLP1 were observed, along with decreased levels of SYN and PSD95, and elevated neuronal death and ROS production in the hippocampus (Jia et al., 2023). Long-term treatment with SS31 reversed these effects. Additionally, SS31 therapy corrected the cognitive deficits found in APP/PS1 transgenic mice. This study found that SS31 reduces ROS and Aβ levels, protects mitochondrial homeostasis and synaptic integrity, and improves behavioral impairments in early-stage AD. These findings suggest that SS31 may serve as a potential pharmacological treatment for managing or reducing the progression of Alzheimer’s disease. Melatonin is a methoxyindole that transmits circadian information about light and darkness (Claustrat and Leston, 2015). It also induces autophagy by decreasing methamphetamine toxicity, which protects against neuronal cell death in the AD brain (Hossain et al., 2021; Nopparat et al., 2023). Furthermore, depending on the phase of autophagy, melatonin can act as either a pro-autophagic signal or an anti-autophagic regulator (Hossain et al., 2019). Additionally, melatonin possesses antioxidant properties that help prevent mitochondrial damage, oxidative stress, and apoptosis. In a Parkinson’s disease model, melatonin can reduce oxidative stress, thereby decreasing mitochondrial breakdown and neuronal loss (Chuang et al., 2016).

There have been many hypotheses that put forth on the possible processes by which senolytics treat neurodegenerative illnesses (Zhu et al., 2017). Nevertheless, it is still not entirely clear how these treatments work (Campisi et al., 2019). Elimination of senescent cells is one strategy that may be used; these cells can cause neuroinflammation and neurodegeneration by the production of proinflammatory cytokines, chemokines, and matrix metalloproteinases. This can be prevented by the removal of these cells (Zhang et al., 2019; Campisi et al., 2019). Restoring the equilibrium between autophagy and apoptosis is another strategy that may be used; this can facilitate the removal of damaged proteins and organelles and stop the formation of hazardous aggregates (Zhang et al., 2019; Wong et al., 2020; Baar et al., 2017). Senescent cells may impede synaptic plasticity and neuronal activity by the production of toxic substances, which can be reversed by senolytics, which have the potential to have direct impacts on neuronal function (Wong et al., 2020; Baar et al., 2017).

In the context of clinical application, the development of senolytic drugs faces several limitations and hurdles. Key concerns include ensuring specificity toward senescent cells to avoid off-target effects, which is crucial for safety and efficacy. Additionally, competition from existing therapies for age-related conditions may hinder market acceptance. High costs associated with novel senolytic treatments could limit patient accessibility (Wong et al., 2023). Furthermore, the heterogeneity of the SASP complicates the identification of effective therapeutic targets (Chaib et al., 2022). Eventually, the need for extensive clinical trials to establish safety and efficacy remains a significant barrier to successful translation into clinical practice (Lelarge et al., 2024).

### Future perspectives

Senolytics have emerged as a promising approach for treating age-related diseases, including neurodegenerative conditions. Even though preclinical and clinical trials of the current senolytic drugs have shown promising results, their possible side effects are still a worry. Henceforth, assessing the senolytic activity of phytochemicals and exploring novel targets for senolysis will be crucial. Phytochemicals are compounds that come from plants and have been shown to have different pharmacological effects, such as slowing the aging process (Iyaswamy et al., 2023; Krishnamoorthi et al., 2023). Several studies have shown that phytochemicals like fisetin, quercetin, and curcumin can slow the aging process (Guan et al., 2024). It has been shown that these phytochemicals can selectively kill off old cells and make mice live longer and healthier. So, testing phytochemicals for their ability to slow the aging process could lead to the discovery of new compounds that could be used as senolytic drugs.

Notably, c current screening techniques include high-throughput library screenings and bioinformatics approaches to identify compounds targeting senescent cell anti-apoptotic pathways (SCAPs) (Zhang et al., 2021). For instance, prodrug strategies can enhance specificity for senescent cells by linking active compounds to moieties that activate in the presence of senescence markers (Zhang et al., 2021). Additionally, machine learning models have been successfully employed to predict senolytic activity from existing data, streamlining the identification process (Smer-Barreto et al., 2023). Future research should focus on integrating these methodologies, emphasizing robust validation in preclinical models to ensure efficacy and safety before clinical application (Chaib et al., 2022; Lelarge et al., 2024). By expanding on these techniques, researchers can better navigate the complexities of senolytic drug discovery and optimize the therapeutic potential of phytochemicals in combating age-related diseases.

Further, besides exploring novel senolytic compounds, identifying new targets for senolysis will be crucial. Current senolytic drugs primarily target the anti-apoptosis pathways activated in senescent cells. Recent studies, on the other hand, have found that senescent cells are also overactive in the inflammasome pathway and the mTOR pathway. Because of this, finding new senolysis targets could lead to the creation of more effective senolysis drugs. Developing methods to detect senescent cells without causing harm to the body is another key goal for the future. Currently, markers like p16INK4a and SA-*β*-Gal) are used to identify senescent cells. However, these methods are invasive and require a tissue biopsy. Developing non-invasive techniques to detect senescent cells could simplify the diagnosis and treatment of age-related diseases.

Overall, the future of senolytics appears promising, especially with the potential discovery of new compounds and targets for senolysis. Additionally, developing non-invasive methods to detect senescent cells could improve the diagnosis and treatment of age-related diseases.

## Conclusion

In summary, targeting cellular senescence holds significant promise as a therapeutic approach for neurodegenerative diseases. The accumulation of senescent cells in the brain has been implicated in the pathogenesis of various neurodegenerative disorders, including AD, PD, ALS, and HD. By selectively eliminating these senescent cells, it may be possible to halt or even reverse the progression of these debilitating conditions. Recent research has shed light on the molecular mechanisms underlying cellular senescence and its role in neurodegeneration. Strategies aimed at disrupting the SASP and promoting the clearance of senescent cells have shown encouraging results in preclinical studies. Furthermore, the development of senolytic drugs that specifically target and eliminate senescent cells has opened new avenues for therapeutic intervention. Despite the promising potential of targeting cellular senescence, several challenges remain. Further research is needed to better understand the complex interplay between senescent cells and the surrounding microenvironment in the brain. Additionally, the long-term safety and efficacy of senolytic therapies need to be carefully evaluated in clinical trials. The field of targeting cellular senescence for the therapy of neurodegenerative diseases is rapidly evolving, with exciting discoveries being made that could revolutionize the treatment of these conditions. By continuing to explore novel therapeutic strategies and innovative approaches, we may 1 day be able to harness the power of cellular senescence to combat neurodegeneration and improve the quality of life for millions of individuals affected by these devastating diseases.
